# ANT1 Activation and Inhibition Patterns Support the Fatty Acid Cycling Mechanism for Proton Transport

**DOI:** 10.3390/ijms22052490

**Published:** 2021-03-02

**Authors:** Jürgen Kreiter, Anne Rupprecht, Sanja Škulj, Zlatko Brkljača, Kristina Žuna, Denis G. Knyazev, Sarah Bardakji, Mario Vazdar, Elena E. Pohl

**Affiliations:** 1 Institute of Physiology, Pathophysiology and Biophysics, Department of Biomedical Sciences, University of Veterinary Medicine, 1210 Vienna, Austria; juergen.kreiter@vetmeduni.ac.at (J.K.); anne.rupprecht@med.uni-rostock.de (A.R.); kristina.zuna@vetmeduni.ac.at (K.Ž.); sarah.bardakji@vetmeduni.ac.at (S.B.); 2 Institute of Pharmacology and Toxicology, Rostock University Medical Center, 18057 Rostock, Germany; 3 Division of Organic Chemistry and Biochemistry, Rudjer Bošković Institute,10000 Zagreb, Croatia; Sanja.Skulj@irb.hr (S.Š.); Zlatko.Brkljaca@irb.hr (Z.B.); Mario.Vazdar@irb.hr (M.V.); 4 Institute of Biophysics, Johannes Kepler University, 4020 Linz, Austria; denis.knyazev@jku.at; 5 Institute of Organic Chemistry and Biochemistry, Czech Academy of Sciences, 16610 Prague 6, Czech Republic

**Keywords:** fatty acid anion transport, proton transport, ADP/ATP carrier protein, mitochondrial transporter, arachidonic acid, long-chain fatty acids

## Abstract

Adenine nucleotide translocase (ANT) is a well-known mitochondrial exchanger of ATP against ADP. In contrast, few studies have shown that ANT also mediates proton transport across the inner mitochondrial membrane. The results of these studies are controversial and lead to different hypotheses about molecular transport mechanisms. We hypothesized that the H^+^-transport mediated by ANT and uncoupling proteins (UCP) has a similar regulation pattern and can be explained by the fatty acid cycling concept. The reconstitution of purified recombinant ANT1 in the planar lipid bilayers allowed us to measure the membrane current after the direct application of transmembrane potential ΔΨ, which would correspond to the mitochondrial states III and IV. Experimental results reveal that ANT1 does not contribute to a basal proton leak. Instead, it mediates H^+^ transport only in the presence of long-chain fatty acids (FA), as already known for UCPs. It depends on FA chain length and saturation, implying that FA’s transport is confined to the lipid-protein interface. Purine nucleotides with the preference for ATP and ADP inhibited H^+^ transport. Specific inhibitors of ATP/ADP transport, carboxyatractyloside or bongkrekic acid, also decreased proton transport. The H^+^ turnover number was calculated based on ANT1 concentration determined by fluorescence correlation spectroscopy and is equal to 14.6 ± 2.5 s^−1^. Molecular dynamic simulations revealed a large positively charged area at the protein/lipid interface that might facilitate FA anion’s transport across the membrane. ANT’s dual function—ADP/ATP and H^+^ transport in the presence of FA—may be important for the regulation of mitochondrial membrane potential and thus for potential-dependent processes in mitochondria. Moreover, the expansion of proton-transport modulating drug targets to ANT1 may improve the therapy of obesity, cancer, steatosis, cardiovascular and neurodegenerative diseases.

## 1. Introduction

In mitochondria, oxidative phosphorylation accounts for ATP production by phosphorylating ADP using proton (H^+^) gradient generated by the respiratory chain proteins (coupling). H^+^ can return to the matrix by alternative pathways (uncoupling): (i) inhibitor-non sensitive basal H^+^ leak (J_B_) and (ii) protein-mediated inhibitor-sensitive proton transport (J_H_) [1,2,3]. J_B_ is sensitive to the membrane potential, mitochondrial inner membrane surface area, the composition of phospholipids and free fatty acids (FA) and was observed in mitochondria of all tissues [1]. Uncoupling proteins (UCP) are implicated in the mediation of J_H_ [4,5,6,7,8,9]. As several tissues such as liver, kidney, skin, and others lack any UCPs under physiological conditions (for review, see [10]), mitochondrial adenine nucleotide translocase (ANT, also cited in the literature as AAC or ADP/ATP carrier) was proposed to provide an alternative pathway for proton transport alongside its well-known function to exchange ADP for ATP [11,12,13,14,15].

The H^+^ transporting function of ANT in the presence of palmitate has been first observed in experiments with isolated mitochondria [16,17]. The addition of free FA to the proteoliposomes reconstituted with purified ANT caused the transmembrane potential (ΔΦ) decrease, which was restored by carboxyatractyloside (CATR) and bongkrekic acid (BA) [18]. In brown-fat mitochondria from mice knockout for UCP1, fatty-acid-induced uncoupling could also be inhibited by CATR [19]. The H^+^ conductance of muscle mitochondria from mice knockout for ANT1 was reported to be half that of wild-type controls [20]. Recently, ANT-mediated H^+^ transport was observed in patched mitoplasts [21]. Although the H^+^ transporting function of ANT1 seems to be accepted, discrepancies in results obtained in various experimental systems led to different views on the proton transport mechanism. In the 1980–1990s, several groups recognized that the proton transport could occur by the flip-flop of the protonated form of long-chain fatty acid (FA) without membrane proteins’ participation [22,23]. However, a fatty acid anion (FA^−^) transport is a rate-limiting step in FA circulation and has to be accelerated by proteins. In 1991, Skulachev proposed the “fatty acid circuit hypothesis”, claiming that proteins such as ANT1 and UCP1 mediate the return of the FA^-^ to the cytosolic side of the membrane, resulting in net proton transport catalyzed by the protein [24]. Our previous results obtained for UCP1-UCP3 can be well described based on the FA cycling model and are consistent with the translocation of FA^−^ at the protein/lipid interface.

In contrast, Bertholet et al. proposed FA to be co-factors in H^+^ transport by ANT based on patch-clamp experiments [21]. In this model, FA is not translocated but stays in one place as a part of the protein translocating pathway, where it is (de-)protonated. This mechanism differed from the mechanism suggested by the same group for UCP1, in which UCP1 was regarded as a FA^–^/H^+^ symporter [25]. Moreover, this model does not necessarily assume direct binding of H^+^ to the FA anion and allows the proton transport in both directions.

Here, we hypothesized that the H^+^-transport mediated by ANT has a regulation pattern similar to UCPs and can be explained by the FA cycling concept. The goals of this study were (i) to investigate the dependence of ANT-mediated H^+^ transport on FA structure, (ii) to estimate ANT-specific H^+^ turnover number, and (iii) to examine whether the specific ANT substrates inhibit H^+^ transport.

## 2. Results

### 2.1. ANT1-Mediated Substrate Transport

To evaluate whether recombinant murine ANT1 was correctly refolded in proteoliposomes, we performed ADP/ATP transport measurements using proteoliposomes initially filled with radioactively labeled ^3^H-ATP [26]. After adding ADP to the bulk solution, we measured the release of ^3^H-ATP with time (Supplementary Figure S2). The determined ADP/ATP exchange rate depends on ANT1 content (k_ANT_ = 5.53 ± 0.74 mmol/min/g) and corresponds well to the reported results for ANT reconstituted into liposomes (Supplementary Table S1) [26,27,28,29].

### 2.2. Basal Proton Leak

**Figure 1:**
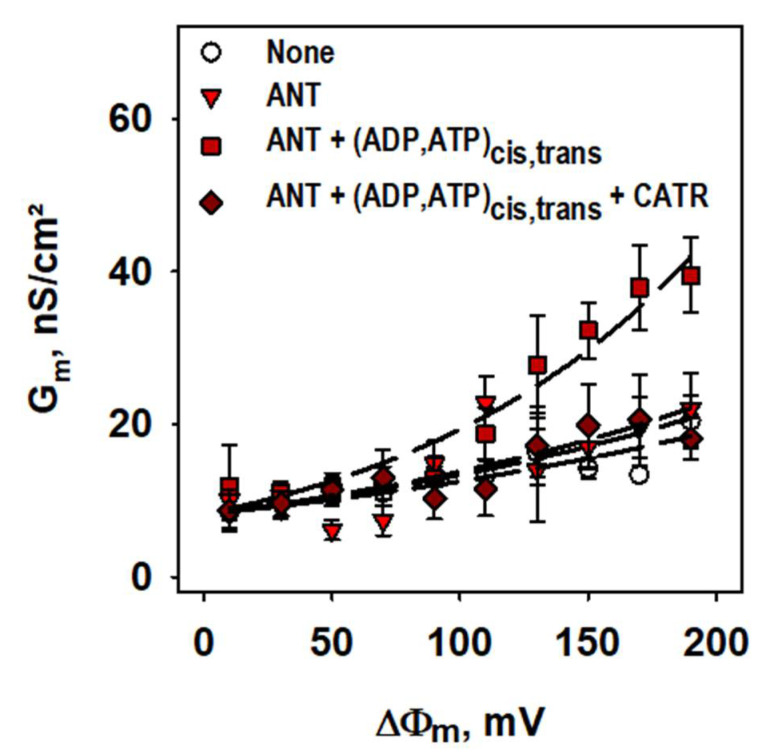
**ANT1 does not contribute to the basal proton leak.** Total membrane conductance (G_m_) was measured at different membrane potentials (ΔΦ) and membrane compositions (s. legend). Planar bilayer membranes were made of 45:45:10 mol % PC:PE:CL. Lipid concentration was 1.5 mg/(mL of buffer solution). Protein concentration measured by BCA assay was 4 µg/(mg of lipid). Buffer contained 50 mM Na_2_SO_4_, 10 mM Tris, 10 mM MES and 0.6 mM EGTA at pH = 7.34 and T = 306 K. ADP, ATP and CATR were added at concentrations 2 mM, 2 mM and 100 µM. Lines represent the least square regression fit of an exponential function to the data. Data are the mean ± SD of at least three independent experiments.

We further investigated the controversially discussed ANT1 involvement in the basal leak [20,21]. For this, we formed planar bilayer lipid membranes from proteoliposomes reconstituted with recombinant ANT1 [26]. Figure 1 demonstrates that the total specific conductances, G_m_ and G_0_, of bilayer membranes made from DOPC, DOPE, and cardiolipin were similar in the presence and absence of ANT1 (G_m_ =10.0 ± 2.0 nS/cm^2^ and G_0_ =8.4 ± 2.3 nS/cm^2^) if no purine nucleotides (PN) were added. The addition of ATP and ADP on both sides of the membrane led to a substantial G_m_ increase, which was directly proportional to the applied membrane potentials, reaching G_m_ = 39.5 ± 4.9 nS/cm^2^ at 190 mV (Figure 1, Supplementary Figure S3 and Supplementary Table S2. This increase vanished after adding the specific inhibitor of ADP/ATP transport—CATR (Figure 1) and can be explained by the electrogenic shift due to ATP/ADP exchange by ANT1. This experiment showed that ANT1 has no measurable impact on the proton leak without FAs.

### 2.3. ANT1-Mediated Proton Transport in the Presence of FA

**Figure 2:**
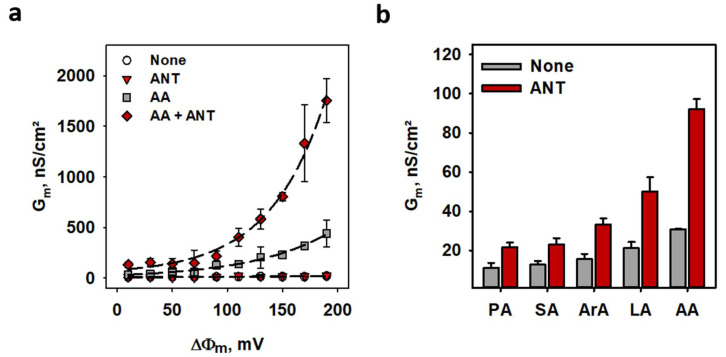
**Fatty acids are required to activate ANT1-mediated proton transport.** (**a**) Total membrane conductance (G_m_) of lipid bilayers in the presence of AA (gray squares), ANT1 (red triangles), ANT1 and AA (dark red diamonds) and in the absence of AA and ANT1 (white circles) at different membrane potentials (ΔΦ_m_). Lines represent the least square regression fit of an exponential function to the data. (**b**) Dependence of total membrane conductance (G_m_) on fatty acid chain length and unsaturation in the presence (red) and absence (gray) of ANT1. PA, SA, ArA, LA, and AA indicate palmitic, stearic, arachidic, linoleic, and arachidonic acids. In all measurements, planar bilayer membranes were made of 45:45:10 mol % PC:PE:CL reconstituted with 15 mol % FA, except indicated otherwise. Lipid concentration was 1.5 mg/(mL of buffer solution). Protein concentration measured by BCA assay was 4 µg/(mg of lipid). The buffer solution contained 50 mM Na_2_SO_4_, 10 mM Tris, 10 mM MES and 0.6 mM EGTA at pH = 7.34 and T = 306 K. Data are the mean ± SD of at least three independent experiments.

The addition of polyunsaturated arachidonic acid (AA) to the membrane in the absence of ANT1 led to a potential-dependent increase in G_m_. It confirms FA’s importance as weak uncouplers, especially at high potentials (G_m_ was one order of magnitude higher at 190 mV) relevant for mitochondrial membranes [30]. The reconstitution of ANT1 in the membrane increased G_m_ in the presence of AA 4-fold (G_m_^ANT, AA^/G_m_^AA^) (Figure 2a). At 190 mV G_m_^ANT, AA^, G_m_^AA^ and G_0_ were equal to 1750 ± 220 nS/cm^2^, 440 ± 135 nS/cm^2^ and 20.4 ± 3.4 nS/cm^2^, respectively (Supplementary Figure S4 and Supplementary Table S2). Notably, ANT1-mediated G_m_ depended on the structure of FAs. It increased with the elongation of FA chain length in order palmitic (PA, 16:0) → arachidic (ArA, 20:0) acid and was the highest by unsaturated AA (20:4) (Figure 2b).

### 2.4. Proton Turnover Number of ANT1

**Figure 3:**
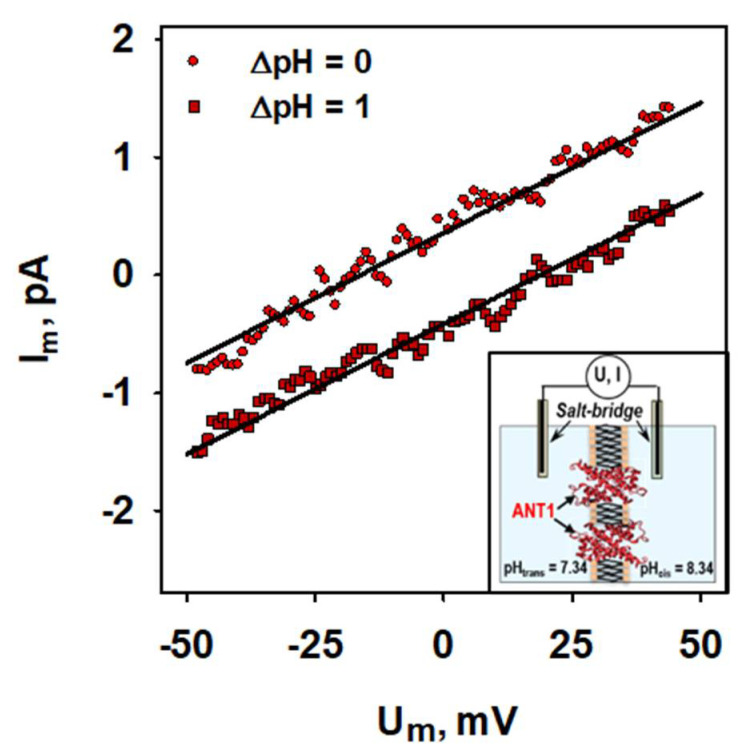
**The proton turnover number of ANT is similar to UCPs.** Representative current-voltage recordings of lipid bilayer membranes reconstituted with ANT1 in the presence (squares) and absence (circles) of ΔpH = 1.0 across the membrane. Lines represent a linear fit to the data. Planar bilayer membranes were made of 45:45:10 mol % PC:PE:CL reconstituted with 15 mol % AA. Buffer contained 50 mM Na_2_SO_4_, 10 mM Tris, 10 mM MES and 0.6 mM EGTA at pH = 7.34 and T = 306 K. Lipid concentration was 1.5 mg/(mL of buffer solution). Protein concentration measured by BCA assay was 4 µg/(mg of lipid). Insert: Experimental setup of the measurements to establish a transmembrane pH gradient.

To determine the H^+^ turnover number of ANT, we recorded current-voltage characteristics in the presence and absence of a transmembrane pH gradient (Figure 3, insert) [31].

To estimate a protein to lipid ratio, we measured the number of fluorescently-labeled ANT per liposome using fluorescence correlation spectroscopy (FCS) [32] (s. Methods and Supplementary Figure S5). By comparing the number of the fluorescent particles in proteoliposomes before (N_ANT, none_ = 1.60 ± 0.01) and after (N_ANT, SDS_ = 13.83 ± 0.04) the addition of 2 % (*v*/*v*) SDS, and assuming one ANT protein per detergent micelle after micellization, we calculated 8.67 ± 0.74 ANT molecules per liposome. The protein to lipid ratio estimated according to Equation (4) was 1:12,000.

From the potential shift and proton/lipid ratio, we then estimated that ANT has a turnover rate of 14.6 ± 2.5 H^+^/s (Figure 3), being similar to those of uncoupling proteins (Supplementary Table S3) [6,7,9,33,34,35].

### 2.5. Inhibition of ANT1-Mediated Proton Transport

**Figure 4:**
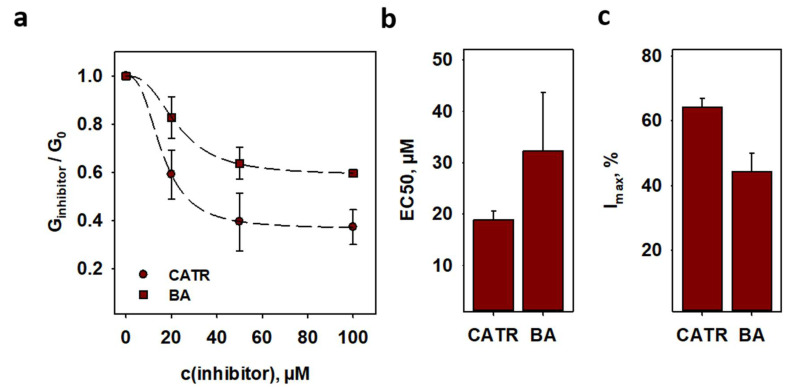
**The ADP/ATP exchange inhibitors inhibit ANT-mediated proton leak.** (**a**) Dose-dependent inhibition of ANT-mediated proton leak by the inhibitors CATR (circles) and bongkrekic acid (BA, triangles) on proton leak (dark red). Lines are a least square regression fit of a sigmoidal function to the data. (**b**) EC50 and (**c**) maximum inhibition I_max_ as fit parameters of (**a**). Planar bilayer membranes were made of 45:45:10 mol % PC:PE:CL reconstituted with 15 mol % AA. Buffer contained 50 mM Na_2_SO_4_, 10 mM Tris, 10 mM MES and 0.6 mM EGTA at pH = 7.34 and T = 306 K. Lipid concentration was 1.5 mg/(mL of buffer solution). Protein concentration measured by BCA assay was 4 µg/(mg of lipid). Data are the mean ± SD of at least three independent experiments.

Specific inhibitors of nucleotide transport lock ANT either in its cytosolic-opened c-side (CATR) or in its matrix-opened m-side (BA) [15] and inhibit both ADP/ATP exchange and FA-mediated proton leak. The comparison of CATR and BA effect on G_m_ (Figure 4a) showed that inhibition by CATR was more effective than by BA. It is displayed by the EC50 values of 18.9 ± 1.8 µM for CATR and 32.3 ± 11.4 µM for BA, respectively (Figure 4b and Supplementary Table S4). Maximum inhibition values (I_max_ = 64.2 ± 2.8% and I_max_ = 44.3 ± 5.7% in the presence of CATR or BA) indicate that ANT conformation in the bilayer is approximately 60% in the c-state and 40% in the m-state in our system (Figure 4c and Supplementary Table S4).

**Figure 5:**
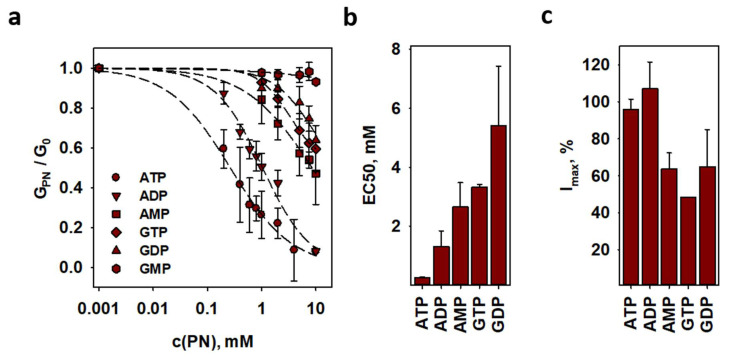
**FA activated proton leak is preferably inhibited by ADP and ATP and maintains the substrate specificity of ANT.** (**a**) Membrane conductance of lipid bilayers reconstituted with AA and ANT1 in the presence of different purine nucleotides. Lines are a least square regression fit of a sigmoidal function to the data. (**b**) EC50 and (**c**) maximum inhibition values as fit function parameters in (**a**). Values for GMP were dropped due to the low effect. For experimental conditions, see Figure 4. Data are the mean ± SD of at least three independent experiments.

To investigate the interdependence of proton transport, activated by FA, and ADP/ATP transport, we measured G_m_ of ANT1-containing lipid bilayers reconstituted with AA in the presence and absence of purine nucleotides (PN). Adenine nucleotides inhibited H^+^ transport much more effectively than guanosine nucleotides (Figure 5a). The EC50 values correlated well with the known narrow substrate specificity of ANT (Figure 5b and Supplementary Table S4) [36]. ATP and ADP fully inhibited G_m_, whereas all other PN decreased G_m_ by 50% (Figure 5c and Supplementary Table S4).

### 2.6. Analysis of the ANT´s Surface Electrostatic Potential using Molecular Dynamic Simulations 

**Figure 6:**
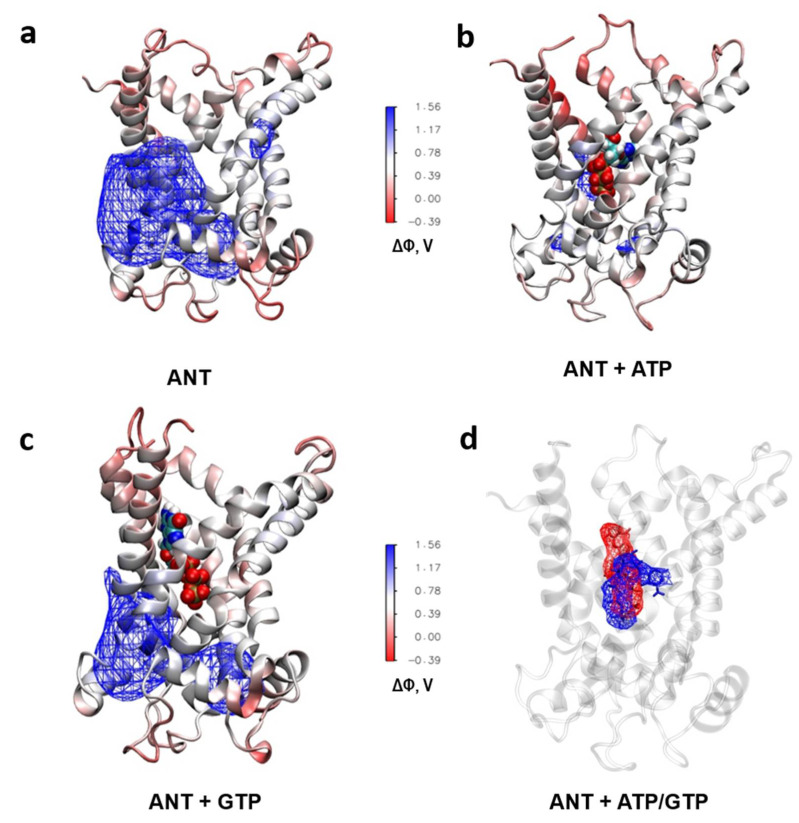
The purine nucleotides ATP and GTP differently modulate the electrostatic surface potential of ANT upon binding. (**a**–**c**) Electrostatic potential (Δ of ANT1 in the absence (**a**) and presence of bound ATP (**b**) and GTP (**c**) calculated by molecular dynamics simulations. The isosurface of the potential of 0.9 V is shown with the wireframe. (**d**) Different average binding location of ATP (blue wireframe) and GTP (red wireframe) in ANT1.

The high similarity of the ANT activation pattern to those of UCP1, UCP2 and UCP3 [6,7,35] leads to the hypothesis that the H^+^ transport can be explained by the FA cycling mechanism. We analyzed the ANT’s surface electrostatic potential in the DOPC bilayers to test whether a possible FA translocation pathway may be localized at the lipid-protein interface. The calculation revealed a large positively charged patch (Figure 6a) that might facilitate FA anion’s sliding alongside the protein. The ATP binding significantly decreased the positive electrostatic potential (Figure 6b) due to its strong screening at the bottom of the cavity [37]. The existence of such a patch would explain the inhibition of H^+^ transport by ATP observed in electrophysiological experiments. GTP has less effect on the positive electrostatic potential (Figure 6c) because of its different orientation in the cavity (Figure 6d) and its “weaker interaction with the hydrophobic pocket that binds the adenine moiety” [36].

## 3. Discussion

We investigated the regulation of H^+^ transport using planar bilayer membranes reconstituted with the recombinant mouse ANT1. This model allowed us (i) to measure membrane conductance at precisely defined lipid and buffer composition, (ii) to apply mitochondria-relevant potentials directly, and (iii) to separate the ANT1-originated effects from the simultaneous effects of other proteins. The latter is a main disadvantage by the interpretation of experiments on (isolated) mitochondria or mitoplasts representing swollen mitochondria lacking an intact outer membrane.

**Figure 7:**
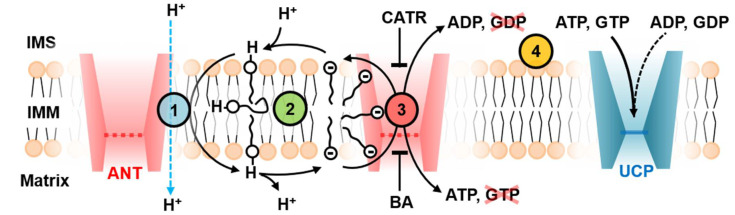
**ANT1 transport features point to the fatty acid cycling mechanism.** The proton transport rate of ANT1 (Table S3) in the presence of fatty acids (FA) is similar to that proposed for UCPs and depends on the FA structure (Circle 1, blue). ANT1 facilitates the FA anion’s transport at the protein-lipid interface, which is supported by the membrane conductance dependence on the FA structure (Circle 2, green). The FA anions slide alongside the electrostatic surface potential of ANT1; its modulation by binding ADP/ATP specific substrates inhibits the FA anion transport (Circle 3, red). The inhibition of ANT1-mediated proton leak is the strongest for the ANT1 substrates—ADP and ATP. It is in contrast to UCPs, in which the triphosphate nucleotides ATP and GTP are the most potent inhibitors (Circle 4, yellow).

We confirmed that ANT1 has a dual function performing H^+^ transport additionally to the substrate transport. Proton transport occurs only in the presence of the long-chain FAs and reveals high sensitivity to the FA chain length and saturation (Figure 7). The data on ANT1 activation showed remarkable similarity to the activation pattern of uncoupling proteins (UCP1-UCP3) [6,7,35]. The dependence of proton transport rate on the FA structure can be explained by the FA cycling model, assuming that the transport of FA anions, which is the rate-limiting step, occurs at the lipid-protein interface as proposed for UCP2 [6].

Although the FA hydrophobicity increases with both chain length and unsaturation, the FA anions occupy similar positions at the lipid-water interface [38]. FA anions should also not further penetrate the ANT1 structure unless a hydrophobic pocket would pull the FA into the ANT1 interior. However, such a membrane-spanning pocket is not found in the ANT structure [39]. 

Whereas the FA activation pattern of the proton transport seems to be similar in ANT and UCPs, the inhibition pattern is not (Figure 7). We explain it by the fact that these proteins transport different substrates. However, all ANT-specific substrates bind at the substrate-binding site in the ANT cavity [39]. As shown by molecular dynamics simulation, after the ATP binding, the electrostatic potential is diminished, and FAs are potentially less attracted to the ANT surface (Figure 6a,b). Since all substrates (ATP^4−^, ADP^3−^, CATR^4−^ and BA^3−^) are similarly charged and bind to the same region, the electrostatic potential of ANT will be significantly altered upon their binding [36,39,40]. GTP has a different orientation when bound to ANT, which has less impact on ANT’s electrostatic potential (Figure 6c,d and Figure 7). The surface electric charge directly correlated with the inhibition potency of PN. This observation strongly supports the data on the binding site competition between PNs and FAs.

Our model is in strong contrast to the model, which proposed that FAs bind within the ANT cavity and act as a co-factor of H^+^ transport [21]. The authors based their model on the experiments showing that non-protonable sulfonated FA failed to induce any transmembrane current in isolated mitoplasts. The use of sulfonated FA is very controversial, as chemical and geometrical properties of the crucial head group are altered compared to the carboxylic head group of native FA. However, the absence of any transient current by non-protonable FA acids is well described by the FA cycling model, in which the net charge transport of H^+^ is impaired by the inability of the sulfonated FA to transport a proton across the membrane [41,42]. Upon FA addition to the mitoplast matrix, Bertholet et al. [21] observed no current in contrast to the FA addition to the cytosolic side, showing that the H^+^ transport is independent of ANT conformation. The authors claimed that FA reaches its putative binding site inside ANT only from the cytosolic side. However, it is questionable how FAs should activate H^+^ transport inside ANT, as protons have to cross at least one salt-bridge network [29], independent of ANT conformation. The FA binding to ANT and subsequently H^+^ binding to the FA in the protein cavity will most probably not provide the energy of roughly 10 kcal/mol to break the strong salt-bridge network [42,43,44,45,46].

The inhibition of H^+^ transport in the FA co-factor model can be clearly described as a competition between FA and purine nucleotides for the binding site that we also observed for UCP1 and UCP3 [7]. However, the FA binding site is not further characterized, and it is not clear if there is a common binding target for the FA anion and the adenine nucleotides and/or specific transport inhibitors. Nevertheless, the dependency on FA chain length and unsaturation would imply a loose binding of FAs inside the protein to account for the different structures, which contrasts the high energy required to break the salt-bridge network. Thus, the model proposed by Bertholet et al. [21] seems to fail in describing our experimental results.

We determined the ANT-specific H^+^ turnover number of 14.6 ± 2.5 H^+^/s, which is similar to turnover numbers determined earlier for UCPs. Besides ANT and UCPs, FA-activated H^+^ leak was also shown for other mitochondrial carriers, including the aspartate/glutamate carrier, dicarboxylate carrier, 2-oxoglutarate carrier, and the phosphate carrier [47,48,49,50]. Thus, it is reasonable to conclude a dual transport function for the before mentioned proteins: (i) the substrate transport to maintain mitochondrial respiration and (ii) the proton transport, which may affect the inner mitochondrial membrane potential. We speculate that these carriers have a similar mechanism of FA-mediated activation of H^+^ transport due to their high homology. Simultaneous activation of several proton transporters could ensure an essential drop in potential. The latter is crucial for regulating potential-dependent processes in mitochondria, such as reactive oxygen species production, cell death, autophagy, protein secretion, metabolic adaptations, and cell signaling [51]. The controlling of the mitochondrial uncoupling can be used to treat several human diseases, such as obesity, cardiovascular diseases, or neurological disorders.

## 4. Methods

### 4.1. Chemicals

Agarose (#3810), KCl (#6781), Na_2_SO_4_ (#8560), 2-(N-morpholino)ethanesulfonic acid (MES, #4256), sodium dodecyl sulfate (SDS, #0183), tris(hydroxymethyl)-aminomethane (Tris, #AE15), chloroform (#AE54) and ethylene glycol-bis(β-aminoethyl ether)-N,N,N′,N′-tetraacetic acid (EGTA, #3054) were purchased from Carl Roth GmbH & Co. KG (Karlsruhe, Germany). Hexane (#296090), hexadecane (#296317), palmitic acid (#P0500), stearic acid (#S4751), arachidic acid (#A3631), linoleic acid (#L1376) and arachidonic acid (#A3611), dimethyl sulfoxide (DMSO, #472301), the purine nucleotides adenine and guanine tri-, di-, and mono-phosphate (ATP, #A2383; ADP, #A2754; AMP, #01930; GTP #G8877; GDP, #G7127; and GMP, #G8377), carboxyatractyloside (CATR, #C4992) and bongkrekic acid (BA, #B6179) were purchased from Sigma-Aldrich (Vienna, Austria). 1,2-dioleoyl-sn-glycero-3-phosphocholine (DOPC, #850375P), 1,2-dioleoyl-sn-glycero-3-phosphoethanolamine (DOPE, #850725P) and cardiolipin (CL, #710335P) came from Avanti Polar Lipids Inc. (Alabaster, AL, USA).

### 4.2. Cloning, Purification and Reconstitution of Murine ANT1

Cloning, purification and reconstitution of murine ANT1 followed a previously established protocol [26]. The protein concentration in proteoliposomes was measured with the Micro BCA^TM^ Protein Assay Kit (Thermo Fisher Scientific, Prod. #23235, Waltham, MA, USA). Protein purity was verified by SDS-PAGE and silver staining (Supplementary Figure S1).

### 4.3. Exchange Rate Measurements of mANT1

ANT-mediated exchange of ADP/ATP was measured radioactively using ^3^H-ATP (Prod. #NET420250UC, Perkin Elmer, Waltham, MA, USA) following the protocol as described elsewhere [26] (Supplementary Figure S2).

### 4.4. Electrophysiological Measurements of mANT1

Planar lipid bilayers were formed from proteoliposomes as described previously [31,52]. FAs were added to the lipid phase before membrane formation. Proper membrane formation was verified by measuring membrane capacitance (C = 0.72 ± 0.05 µF/cm^2^), which is independent of the presence of protein, FA and inhibitor. Current-voltage (I-U) measurements were performed with a patch-clamp amplifier (EPC 10USB, Werner Instruments, Holliston, MA, USA). The specific total membrane conductance (G_m_) at 0 mV was obtained as the slope of a linear fit of the experimental data at applied voltages from −50 mV to + 50 mV and normalized to the membrane area in cm^2^. Purine nucleotides (solved in distilled water, pH = 7.34) and ANT-specific inhibitors BA and CATR (solved in DMSO) were added to the buffer solution before forming bilayer membranes. The concentrations of each substrate are indicated in the figure legends. Membrane conductance expressed in relative units was calculated according to [7].

Measurement and calculation of H^+^ turnover rate of ANT followed the established protocol [31]. The addition of Tris increased the pH value of the buffer solution on the cis side of the membrane to a value of pH = 8.34.

### 4.5. Fluorescence Correlation Spectroscopy (FCS)

The average number of ANT1 per liposome was measured using FCS [32,53]. In brief, proteoliposomes obtained after reconstitution of the ANT1 were extruded using a Mini-Extruder system (Avanti Polar Lipids Inc., Alabaster, AL, USA) with a membrane nanopore filter with a pore diameter of 100 nm (Avestin Europe GmbH, Mannheim, Germany, LFM-100). ANT1 was labeled with ATTO 488-maleimide [54] (Sigma-Aldrich, Vienna, Austria; 28562-1MG-F). We used size exclusion chromatography (Sephadex^®^ G-50 Superfine, Merck, Vienna, Austria; GE17-0041-01) to remove the unbound dye. The average residence time, τ_D,_ and ANT1- containing proteoliposomes number in the focal volume were derived from the autocorrelation function (G(τ)) of the temporal fluorescence signal (Supplementary Figure S5). To measure the signal, a commercial laser scanning microscope (LSM 510 META/ConfoCor 3, Carl Zeiss, Jena, Germany) equipped with avalanche diodes and a 40× water immersion objective was used. The standard model for two-component free 3D diffusion was applied [55]:

G(τ) = 1 + 1/(n (1+ τ/τ_D_)) (1)

where the number of fluorescent particles, n, in the detection volume, V_eff_, was determined as n = V_eff_ C, where C is the particle concentration. The diffusion coefficient (D) was determined as D = ω^2/^4τ_D_, where ω = 0.16 µm is the diameter of the confocal volume cross-section as determined from the calibration experiments.

Dissolving the liposomes with 2% (*v*/*v*) SDS was expected to increase the particle number if liposomes contained more than one ANT1. The average number of ANT1 per liposome, <N_ANT1_*>*, was obtained from the ratio of the particle number per confocal volume after and before the addition of SDS.

The protein per lipid ratio (ρ) was estimated by:

(2) $$\rho =\frac{N_{\mathrm{ANT}1}}{N_{\mathrm{Lipids}}}={\frac{<N_{\mathrm{ANT}1}>}{<N_{\mathrm{Lipids}}>}|}_{\mathrm{Liposomes}}$$

The average number of lipids per liposomes (<N_Lipids_>) is calculated by the ratio of the surface area of the liposome with radius r and the average area per lipid (A_L_ ≈ 0.6 nm^2^) of a membrane containing DOPC, DOPE and CL [56,57]:

(3) $$<N_{\mathrm{Lipids}}>=2\frac{4{\pi r}^{2}}{A_{L}}$$

Thus, Equation (2) gives:

(4) $$\rho =<N_{\mathrm{ANT}1}>\frac{1}{2}\frac{A_{L}}{4{\pi r}^{2}}$$

### 4.6. Molecular Dynamics Simulations

We performed all-atom molecular dynamics (MD) simulations of ANT1 protein in a 1,2-dioleoyl-sn-glycero-3-phosphocholine (DOPC) bilayer. Residues (residue 1 and residues 294–297) missing from the crystal structure of ANT1 (PDB code: 1okc) [40] without CATR were added using Modeller 9 [58] and implemented into the DOPC bilayer using CHARMM-GUI (http://www.charmm-gui.org/ (accessed on 7 January 2021)) [59,60,61]. Three system setups were prepared—the wild-type ANT1, the wild-type ANT1 with ATP^4−^ bound in the cytosolic-open state (c-state) [43], as well as GTP^4−^ bound in the same position. All simulation boxes contained ANT1 protein (with a total charge of +19), 73 DOPC molecules per leaflet (146 per system), ~11,500 water molecules, and the necessary number of Cl^-^ anions to neutralize the net charge, depending on whether ATP^4−^ or GTP^4−^ are added to the system. All systems were first minimized and equilibrated in six steps using the CHARMM-GUI protocol [62] and then simulated for a further 100 ns without any restraints with a 2 fs time step in a periodic rectangular box of 7.9 nm × 7.9 nm × 9.4 nm using the isobaric-isothermal ensemble (NPT) and periodic boundary conditions in all directions at T = 310 K, maintained via Nosé–Hoover thermostat [63] independently for the DOPC, water/ions and protein subsystems with a coupling constant of 1.0 ps^−1^. The pressure was set to 1.013 bar and controlled with a semi-isotropic Parrinello-Rahman barostat [64] with a time constant for pressure coupling of 5 ps^−1^. Long-range electrostatics were calculated using the particle-mesh Ewald (PME) method [65] with real space Coulomb interactions cut off at 1.2 nm using a Fourier spacing of 0.12 nm and a Verlet cut-off scheme. All simulated systems were described by the CHARMM36m force field [66]. The electrostatic potential maps of all systems were calculated using VMD’s PMEPOT plugin [67]. All simulations were run with the GROMACS 5.1.4 software package [68] and visualized with the VMD molecular graphics program [69].

### 4.7. Statistics

Data analysis and fitting of electrophysiological measurements were performed using Sigma Plot 12.5 (Systat Software GmbH, Erkrath, Germany) and displayed as mean ± SD of at least three independent measurements.

## Data Availability

The datasets generated and/or analyzed during this study are available from the corresponding authors on reasonable request.

## Supplementary Materials

The following are available online at https://www.mdpi.com/1422-0067/22/5/2490/s1.
