# Supplementary material for: ANT1 Activation and Inhibition Patterns Support the Fatty Acid Cycling Mechanism for Proton Transport
Source: Int J Mol Sci. 2021 Mar 2;22(5):2490. doi: 10.3390/ijms22052490 (PMC7958136; doi:10.3390/ijms22052490)
Supplement: Supplementary file 1 [file ijms-22-02490-s001.zip › ijms-1087802 suppl/210108_Suppl_Figures_ANT_final.pdf]

ANT1 activation and inhibition patterns support the fatty acid cycling mechanism for the proton transport

Jürgen Kreiter[1], Anne Rupprecht[1,2], Sanja Škulj[3], Zlatko Brkljača[3], Kristina Žuna[1], Denis G. Knyazev[4], Sarah Bardakji[1], Mario Vazdar[3,5], Elena E. Pohl[1,*]

[1]Institute of Physiology, Pathophysiology and Biophysics, Department of Biomedical Sciences, University of Veterinary Medicine, 1210 Vienna, Austria
[2]Institute of Pharmacology and Toxicology, Rostock University Medical Center, 18057 Rostock, Germany
[3]Division of Organic Chemistry and Biochemistry, Rudjer Bošković Institute, 10000 Zagreb, Croatia
[4]Institute of Biophysics, Johannes Kepler University Linz, 4020 Linz, Austria
[5]Institute of Organic Chemistry and Biochemistry, Czech Academy of Sciences, Flemingovo nám. 2, 16610 Prague 6, Czech Republic

Supplementary Figures

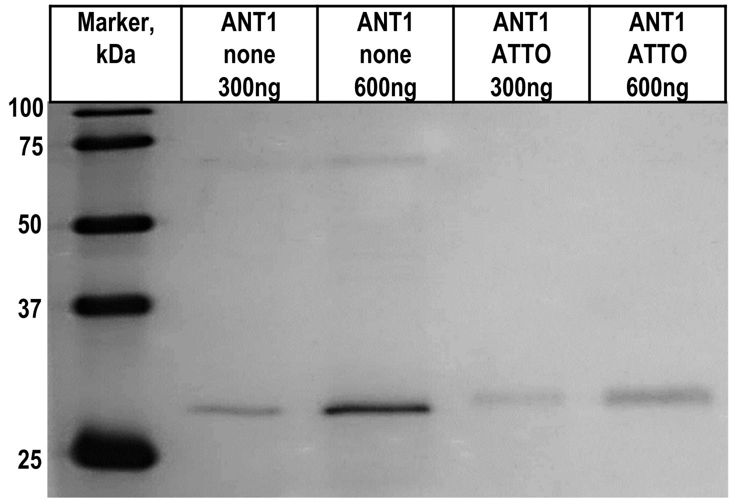

Supplementary Fig. 1. Representative silverstaining of murine ANT1.
For quality control, 300 ng or 600 ng of proteoliposomes were loaded onto a 15% acrylamide gel and SDS-PAGE was conducted. Subsequently, proteins were visualized by silver staining. Precision Plus ProteinT Dual Color Standard (Bio-Rad) was loaded as a molecular weight marker. ANT1 shown in the third and forth lanes was stained by fluorescent dye ATTO.

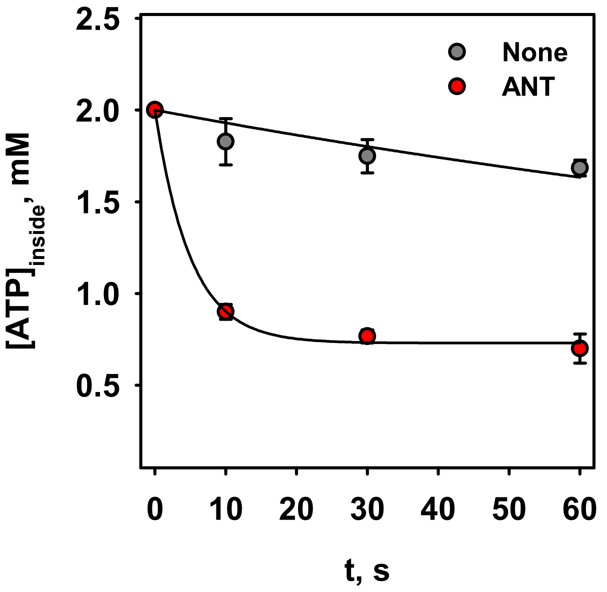

Supplementary Fig. 2. ADP/ATP exchange mediated by the recombinant murine ANT1 reconstituted in proteoliposomes.

Time course of the ^{3}H-ATP concentration inside empty liposomes (grey) and ANT1-containing liposomes (red) after the addition of 2 mM ADP to the buffer solution at t = 0 s. Lines are the least-square fit of an exponential function to the data. The initial concentration of ^{3}H-ATP inside liposomes was 2 mM. In all measurements, membranes were made of 1 mg/ml lipid mixture (DOPC:DOPE:CL=45:45:10 mol%). Protein concentration estimated by BCA assay was 1.7 µg/(mg of lipid). Buffer solution contained 50 mM Na_2SO_4, 10 mM Tris, 10 mM MES and 0.6 mM EGTA at pH = 7.34 and T = 295 K. Data are displayed as the mean ± SD of at least three independent measurements.

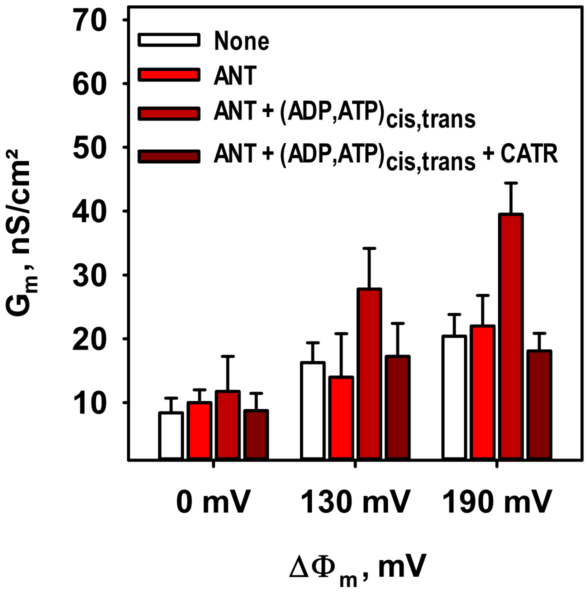

Supplementary Fig. 3. Dependence of ANT1- mediated ADP/ATP exchange on the transmembrane potential ($\Delta\Phi_m$).

Total membrane conductance (G_m) of lipid bilayers in the absence (first bar) and presence of ANT1 (second bar), ANT1 and 2 mM ATP and 2 mM ADP in the buffer solution (third bar) and ANT1 and 2 mM ATP, ADP and 100 µM CATR (fourth bar) in the buffer solution evaluated at $\Delta\Phi_m$ - 0, 130 and 190 mV. Values at 0 mV were deduced from a linear fit to current-voltage recordings from -50 to + 50 mV.

Experimental conditions – as described in Suppl. Fig. 2. Protein concentration estimated by BCA assay was 4 µg/(mg of lipid). Data are displayed as the mean ± SD of at least three independent measurements.

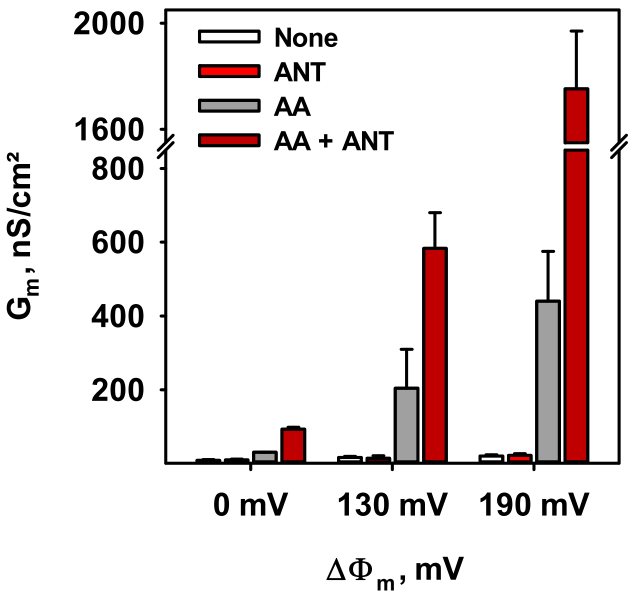

Supplementary Fig. 4. Dependence of ANT1-mediated proton transport on the transmembrane potential ($\Delta\Phi_m$).

Total membrane conductance (G_m) of lipid bilayers in the presence of ANT (light red), AA (grey) and ANT and AA (dark red) and in the absence of ANT and AA (white) evaluated at $\Delta\Phi_m$ - 0, 130 and 190 mV. Values at 0 mV were deduced from a linear fit to current voltage recordings from -50 to + 50 mV.

Experimental conditions – as described in Suppl. Fig. 2. Protein concentration estimated by BCA assay was 4 µg/(mg of lipid). Data are displayed as the mean ± SD of at least three independent measurements.

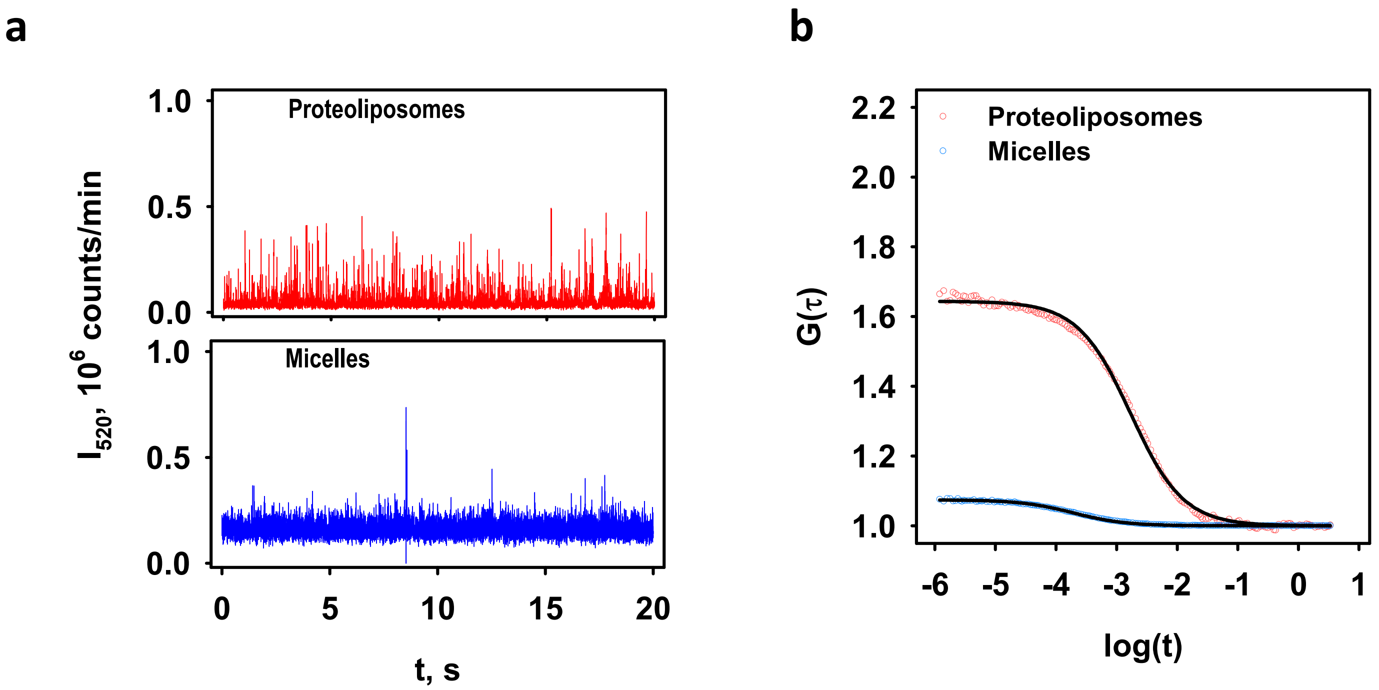

Supplementary Fig. 5. Fluorescence correlation spectroscopy of fluorescently labelled ANT in proteoliposomes and after solubilizing into SDS.

a, Time course of the fluorescence intensity of fluorescently labelled ANT1 in proteoliposomes (red, top) and solved in 2% (v/v) SDS (blue, bottom).

b, Autocorrelation function G(τ) of the temporal signal of fluorescently labeled ANT1 in proteoliposomes (red) and solubilization in 2% (v/v) SDS (blue).

In all measurements, proteoliposomes were made of 45:45:10 mol% DOPC:DOPE:CL and the lipid concentration was 4 mg/ml. Protein concentration measured by the BCA assay was 23.2 μg/ml. The buffer contained 50 mM Na_2SO_4, 10 mM Tris, 10mM MES, 0.6 mM EGTA and 10% (v/v) glycerol at pH = 7.34 and R.T.
