# Supplementary material for: ANT1 Activation and Inhibition Patterns Support the Fatty Acid Cycling Mechanism for Proton Transport
Source: Int J Mol Sci. 2021 Mar 2;22(5):2490. doi: 10.3390/ijms22052490 (PMC7958136; doi:10.3390/ijms22052490)
Supplement: Supplementary file 1 [file ijms-22-02490-s001.zip › ijms-1087802 suppl/210108_Suppl_Tables_ANT_final.pdf]

ANT1 regulation supports the fatty acid cycling mechanism for the proton transport

Jürgen Kreiter[1], Anne Rupprecht[1,2], Sanja Škulj[3], Zlatko Brkljača[3], Kristina Žuna[1], Denis G. Knyazev[4], Sarah Bardakji[1], Mario Vazdar[3,5], Elena E. Pohl[1,*]

[1]Institute of Physiology, Pathophysiology and Biophysics, Department of Biomedical Sciences, University of Veterinary Medicine, Vienna, Austria
[2]Institute of Pharmacology and Toxicology, Rostock University Medical Center, Rostock, Germany
[3]Division of Organic Chemistry and Biochemistry, Rudjer Bošković Institute, Zagreb, Croatia
[4]Institute of Biophysics, Johannes Kepler University Linz, Linz, Austria
[5]Institute of Organic Chemistry and Biochemistry, Czech Academy of Sciences, Flemingovo nám. 2, 16610 Prague 6, Czech Republic

Supplementary Tables

Supplementary Table 1: Overview of ADP/ATP exchange rate of ANT reconstituted into liposomes described in literature.

Protein	$k_{ADP/ATP}$, mmol/min/g	Nucleotides	T, K	Technique	Ref.
Isolated ANT from beef heart mitochondria	0.05 – 0.2	$ADP/ATP_{internal}$ = 5 mM<br>$ADP/ATP_{external}$ = 10 - 100 μM	296	Radioactivity	[1]
Yeast ANT2	3.9 – 8.3	$ADP/ATP_{internal}$ = 20 mM<br>$ADP/ATP_{external}$ = 100 μM	283	Radioactivity	[2]
Yeast ANT2	10.7	$ADP_{internal}$ = 5 mM<br>$ADP_{external}$ = 1.5 μM	296	Radioactivity	[3]
Recombinant murine ANT1	3.49 ± 0.41	$ADP_{internal}$ = 2 mM<br>$ADP_{external}$ = 2 mM	296	Fluorescence	[4]
	2.90 ± 0.47			Radioactivity	
Recombinant murine ANT1	5.53 ± 0.74	$ADP_{internal}$ = 2 mM<br>$ADP_{external}$ = 2 mM	296	Radioactivity	In this study

[1] Kraemer, R., & Klingenberg, M. (1982). Electrophoretic control of reconstituted adenine nucleotide translocation. Biochemistry, 21(5), 1082-1089.

[2] Heidkämper, D., Müller, V., Nelson, D. R., & Klingenberg, M. (1996). Probing the role of positive residues in the ADP/ATP carrier from yeast. The effect of six arginine mutations on transport and the four ATP versus ADP exchange modes. Biochemistry, 35(50), 16144-16152.

[3] King, M. S., Kerr, M., Crichton, P. G., Springett, R., & Kunji, E. R. (2016). Formation of a cytoplasmic salt bridge network in the matrix state is a fundamental step in the transport mechanism of the mitochondrial ADP/ATP carrier. Biochimica et Biophysica Acta (BBA)-Bioenergetics, 1857(1), 14-22.

[4] Kreiter, J., Beitz, E., & Pohl, E. E. (2020). A Fluorescence-Based Method to Measure ADP/ATP Exchange of Recombinant Adenine Nucleotide Translocase in Liposomes. Biomolecules, 10(5), 685.

Supplementary Table 2: Total membrane conductance at 0, 130 and 190 mV.
Respective values as shown in Suppl. Fig. 3 and 4.

	G_m, nS/cm²					
**$\Delta\Phi_m$, mV**	**None**	**ANT**	**ANT + (ADP,ATP)**	**ANT +(ADP,ATP) + CATR**	**AA**	**ANT**
**0**	8.41 ± 2.31	10.0 ± 2.0	11.8 ± 5.5	8.78 ± 2.69	30.8 ± 0.3	93.3 ± 5.1
**130**	16.3 ± 3.1	14.0 ± 6.8	27.8 ± 6.4	17.2 ± 5.2	204 ± 106	584 ± 97
**190**	20.4 ± 3.4	22.0 ± 4.8	39.5 ± 4.9	18.1 ± 2.7	440 ± 135	1750 ± 220

Supplementary Table 3: Overview of fatty acid activated H^+ turnover number of UCP and ANT reconstituted into proteoliposomes

Fatty acids are: AA – arachidonic acid (C20:4), LA – lauric acid (C12:0), OA – oleic acid (C18:1) and PA - palmitic acid (C16:0). * Values are recalculated; ** 6-methoxy-N-(3-sulfopropyl)quinolinium

Protein	k_{H+} ,s^{-1}	Fatty acid	T, K	Measurement system	Ref.
Isolated hamster UCP1	2 - 10	n.a.	284	Proteoliposomes subjected to a pH electrode	[1]
Recombinant rat UCP1, human UCP2 and human UCP3	5.3 – 15.9*	40 µM PA	n.a.	Proteoliposomes loaded with SPQ** fluorescence dye	[2]
Isolated hamster UCP1	14 ± 5	14.5 mol% OA	295	Planar lipid bilayer with transmem-brane pH gradient	[3]
Recombinant human UCP2	4.8	15 mol% AA	310		[4]
Recombinant human UCP2	0.85 – 2.08*	100 µM LA	298	Proteoliposomes loaded with SPQ** fluorescence dye	[5]
Recombinant human UCP4	0.35 – 0.58*	100 µM LA	298		
Recombinant human UCP5	1.39 – 2.67*	100 µM LA	298		
Recombinant murine UCP3	2.56 ± 0.79	15 mol% AA	305	Planar lipid bilayer with transmem-brane pH gradient	[6]
Recombinant murine UCP1	5.56 ± 0.38	15 mol% AA	305		[7]
Recombinant murine UCP3	4.10 ± 0.71	15 mol% AA	305		
Recombinant murine ANT1	14.6 ± 2.5	15 mol% AA	305	Planar lipid bilayer with transmem-brane pH gradient	In this study

References:

[1] Klingenberg, M., & Winkler, E. (1985). The reconstituted isolated uncoupling protein is a membrane potential driven H+ translocator. The EMBO journal, 4(12), 3087-3092.

[2] Jabůrek, M., Vařecha, M., Gimeno, R. E., Dembski, M., Ježek, P., Zhang, M., ... & Garlid, K. D. (1999). Transport function and regulation of mitochondrial uncoupling proteins 2 and 3. Journal of Biological Chemistry, 274(37), 26003-26007.

[3] Urbánková, E., Voltchenko, A., Pohl, P., Ježek, P., & Pohl, E. E. (2003). Transport kinetics of uncoupling proteins analysis of UCP1 reconstituted in planar lipid bilayers. Journal of Biological Chemistry, 278(35), 32497-32500.

[4] Beck, V., Jaburek, M., Demina, T., Rupprecht, A., Porter, R. K., Jezek, P., & Pohl, E. E. (2007). Polyunsaturated fatty acids activate human uncoupling proteins 1 and 2 in planar lipid bilayers. The FASEB Journal, 21(4), 1137-1144.

[5] Hoang, T., Smith, M. D., & Jelokhani-Niaraki, M. (2012). Toward understanding the mechanism of ion transport activity of neuronal uncoupling proteins UCP2, UCP4, and UCP5. Biochemistry, 51(19), 4004-4014.

[6| Macher, G., Koehler, M., Rupprecht, A., Kreiter, J., Hinterdorfer, P., & Pohl, E. E. (2018). Inhibition of mitochondrial UCP1 and UCP3 by purine nucleotides and phosphate. Biochimica et Biophysica Acta (BBA)-Biomembranes, 1860(3), 664-672.

[7] Kreiter, J., & Pohl, E. E. (2019). A micro-agar salt bridge electrode for analyzing the proton turnover rate of recombinant membrane proteins. JoVE (Journal of Visualized Experiments), (143), e58552.

Supplementary Table 4: IC50 and maximum inhibition of the interaction of CATR, BA and PN with ANT.

Respective values deduced as fitting parameters in Fig. 4 and 5.

Inhibitor	EC50, mM	Maximum inhibition, %
CATR	0.019 ± 0.002	64.2 ± 2.8
BA	0.032 ± 0.011	44.3 ± 5.7
ATP	0.250 ± 0.034	95.7 ± 5.7
ADP	1.310 ± 0.537	107 ± 15
AMP	2.657 ± 0.817	63.7 ± 8.7
GTP	3.324 ± 0.093	48.4 ± 0.1
GDP	5.408 ± 2.008	65.0 ± 20.0
GMP	> 10	n.a.
